# The Role of Innate Cells During Alphavirus Chikungunya Infection

**DOI:** 10.3390/v17111469

**Published:** 2025-11-01

**Authors:** Juliane Santos de França da Silva, Livian Maria Silva dos Santos, Célio Valdevino Ferreira Junior, Nathalie de Sena Pereira, Juliana Navarro Ueda Yaochite, Valter Ferreira de Andrade Neto, Paulo Marcos da Matta Guedes, Rafael Freitas De Oliveira França, Ramayana Morais de Medeiros Brito, Manuela Sales Lima Nascimento

**Affiliations:** 1 Department of Microbiology and Parasitology, Biosciences Center, Federal University of Rio Grande do Norte, Natal 59078-970, Rio Grande Do Norte, Brazil; 2 Faculty of Pharmacy, Dentistry & Nursing, Federal University of Ceará, Fortaleza 60355-636, Ceará, Brazil; 3 Laboratory of Translational Medicine, Oswaldo Cruz Foundation FIOCRUZ, Ribeirão Preto 21040-900, São Paulo, Brazil; 4 Division of Biomedical Science and Biochemistry, Research School of Biology, The Australian National University, Canberra, ACT 2600, Australia

**Keywords:** Chikungunya virus, innate immunity, fibroblasts, IFN-type I, innate cells, arthralgia, immunopathogenesis

## Abstract

Alphavirus chikungunya (CHIKV) is an arthropod-borne alphavirus of the *Togaviridae* family, transmitted primarily by *Aedes aegypti* and *Ae. albopictus* mosquitoes. CHIKV infection often results in debilitating manifestations that compromise quality of life and generate significant socioeconomic impacts. Recurrent epidemics in tropical and subtropical regions underscore the urgent need to better understand the host immune responses and their contribution to disease outcome. CHIKV establishes infection by overcoming the host’s initial immunological barriers. Innate immune cells, including fibroblasts, dendritic cells, macrophages, monocytes, neutrophils and natural killer (NK) cells, are among the first to respond to infection, ensuring a rapid antiviral defense and supporting the development of adaptive immune responses. However, excessive release of inflammatory mediators and prolonged infiltration of innate cells into joint tissues contribute to disease chronicity and the persistence of arthralgia. In this review, we provide a comprehensive synthesis of current evidence on innate cells that serve as targets for CHIKV infection, highlighting mechanisms that promote effective antiviral defense as well as those responsible for pathological inflammation and chronic disease and identifying key gaps that remain to be addressed.

## 1. Introduction

**Figure 1:**
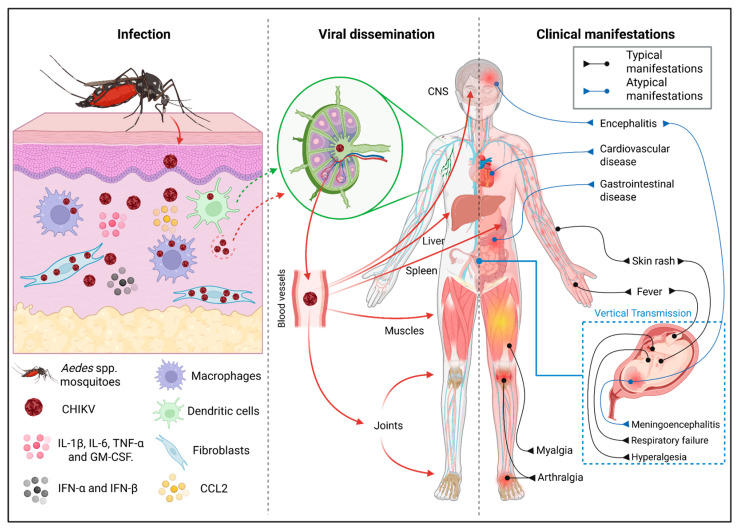
Infection, dissemination and clinical manifestations of Chikungunya fever. CHIKV is transmitted through the bite of *Aedes* mosquitoes, entering the skin and replicating in resident cells such as fibroblasts and Langerhans cells, as well as in macrophages recruited to the tissue. The infection induces an inflammatory and antiviral microenvironment enriched in IL-1β, IL-6, TNF-α, GM-CSF, type I interferons (IFN-α/β) and chemokines (e.g., CCL2). The virus may reach the draining lymph nodes via infected Langerhans cells, while macrophages contribute to viremia by increasing the amount of viral particles that directly disseminate to the lymph nodes. From the lymph nodes, CHIKV spreads into the bloodstream, reaching organs such as the liver, spleen, musculoskeletal tissues, joints and, less frequently, the central nervous system (CNS). The disease results in typical clinical manifestations, including fever, myalgia and intense, often persistent arthralgia. In addition, atypical presentations involving the CNS, gastrointestinal and cardiovascular systems are also reported. Created in BioRender. Silva, J.S.d.F.d. et al. (2025) https://app.biorender.com/illustrations/680fc610a7686a708e2b73be.

Alphavirus chikungunya, the rename of Chikungunya virus (referred to here as CHIKV) [1], is an arthritogenic arbovirus, belonging to the *Togaviridae* family and *Alphavirus* genus and circulating mostly in tropical and subtropical countries; the virus is transmitted to humans by *Aedes aegypti* (*Ae. aegypti*) or *Aedes albopictus* (*Ae. albopictus*) mosquitoes during blood feeding (Figure 1). CHIKV was first identified in humans in 1952, and the infection is commonly characterized by fever, skin rash, myalgia and intense joint pain [2,3] (Figure 1). Atypical clinical manifestations, such as cardiovascular disease, gastrointestinal involvement and central nervous system (CNS) complications, have also been reported, as well as vertical transmission, which can result in severe neonatal outcomes, including encephalitis and respiratory failure [4,5,6,7] (Figure 1).

Previously considered a neglected tropical virus with sporadic outbreaks, CHIKV now has a significant impact on global health. Since 2004, the autochthonous CHIKV transmission has spread to approximately 119 countries across Asia, Africa, the Americas and parts of Europe and Oceania [2,8]. Approximately 35 million infections occur annually, and over 2.8 billion people live in areas with high risk of transmission [9]. In 2024, more than 460,000 suspected cases were registered worldwide [3]. Countries like Brazil, India, Thailand and those in East Africa have experienced recurrent outbreaks, with high morbidity and significant public health costs [10,11]. Additionally, the co-circulation of CHIKV with other arboviruses complicates surveillance and diagnostic efforts [12]. In such scenarios, the World Health Organization (WHO) launched in 2022 the Global Arbovirus Initiative for *Aedes*-borne arboviral diseases, aiming to strengthen preparedness and response to mitigate the ongoing risk of arboviral epidemics, focusing mainly on Chikungunya, Dengue, urban Yellow fever and Zika viruses [3].

Although the fatality rate of CHIKV infection is relatively low (~0.3%), infection can result in debilitating clinical manifestations, causing arthralgia in 90% of cases in the acute phase, which can turn into chronic arthralgia that persists for months or even years. The chronic phase affects about 44% of infected individuals and compromises quality of life, reduces work capacity and generates significant economic impacts [13,14]. There is a strong correlation between chronic chikungunya arthritis and the female sex, the number of first-affected joints and the initial pain level [15]. Furthermore, systematic review and meta-analysis showed that chikungunya mortality is greater in older men with chronic illnesses [16].

As no antiviral treatments are available, symptomatic management and supportive care remain the primary approaches [17]. The US Food and Drug Administration (FDA) licensed the first live-attenuated chikungunya vaccine (IXCHIQ) in November 2023. Prior to this, there were around 35 candidates in different stages of research in the chikungunya vaccine pipeline [2]. However, the FDA suspended IXCHIQ’s U.S. licensing on 22 August 2025. According to the US FDA’s Center for Biologics Evaluation and Research, the vaccination appears to cause Chikungunya-like illness, raising serious safety concerns. Over 20 significant adverse events that were compatible with Chikungunya-like disease were observed, and one encephalitis fatality was directly linked to the IXCHIQ vaccination. Additionally, confirmatory clinical trials have not yet shown the vaccine’s effectiveness [18].

Differences in cellular tropism have been documented for distinct alphaviruses. Arthritogenic alphaviruses such as Chikungunya virus (CHIKV), Ross River virus (RRV) and O’nyong-nyong virus (ONNV) primarily target fibroblasts, macrophages and musculoskeletal tissues, leading to strong local inflammatory responses and joint-associated pathology. In contrast, encephalitic alphaviruses such as Eastern and Venezuelan equine encephalitis viruses (EEEV and VEEV) exhibit a distinct tropism for neuronal and glial cells, often coupled with limited replication in myeloid cells [19,20,21]. Upon infection, CHIKV is known to target different human cell types including epithelial and endothelial cells, primary monocytes and monocyte-derived macrophages (MDMs) [22]. CHIKV triggers pathogenic responses by overcoming the host’s main immunological barriers, being able to replicate within resident cells near the site of inoculation, such as fibroblasts, macrophages and Langerhans cells, then initiating a coordinated replication cycle that ensures viral propagation and dissemination within the host [23,24,25,26] (Figure 1).

The innate immunity represents the host’s first line of defense against invading pathogens. Its activation has a major role in initiating a rapid and broad antiviral response, also triggering an effective adaptive immunity against CHIKV. However, an excessive production of inflammatory mediators contributes significantly to the development of clinical manifestations observed during the acute phase of infection, including fever, malaise and joint inflammation [27]. Moreover, persistent activation of innate immune pathways and a sustained infiltration of inflammatory cells into the joint tissues are key contributing factors to the development of chronic arthritis, a condition observed in over 40% of CHIKV patients, even after viral clearance [15,28,29,30].

Therefore, understanding the response of the first cells involved in CHIKV response is essential for elucidating and maybe modulating the pathophysiology of the disease. In this context, this review synthesizes and discusses current evidence on the innate cells that are targets of CHIKV and how they contribute to effective viral clearance or excessive inflammation and chronic disease manifestation. By outlining target cells and cellular components of the innate immune response to CHIKV, this review can shed light on future research perspectives and the development of novel target strategies to mitigate the infection impact over the population at risk.

## 2. Search Strategy

This review was conducted through a comprehensive literature search in the PubMed, Scopus, Web of Science and Google Scholar databases, covering the period from June to October of 2025. Only articles published using English were considered, with no restrictions on the year of publication. The search strategy employed Boolean operators including AND, OR and NOT to combine the following keywords: “*Chikungunya virus*”, “*Chikungunya immunopathogenesis*”, “*Immune cells*”, “Macrophages/Monocytes”, “Dendritic cells”, “Fibroblasts”, “Neutrophils”, “Cytotoxic innate cells”, “NK cells”, “Innate immunity”, “Chikungunya disease”, “Disease severity”, “Alphavirus Chikungunya”, “Aedes aegypti saliva” “mosquito saliva”, “Vector-host interactions”. The studies reporting data on the role of innate immune cells during Chikungunya infection were manually screened by the authors, and those considered the most relevant were included in this review.

## 3. Fibroblasts as Central Hubs in CHIKV Infection

Following CHIKV inoculation into the host’s skin, innate immune cells carry out several functions that not only help to control viremia but also shape the inflammatory response throughout the infection [31,32,33,34]. Interestingly, fibroblasts emerge as central players during the infection establishment by combining three critical functions: they act as primary viral targets, actively contribute to the local inflammatory milieu and serve as long-term viral reservoirs, thereby linking acute viral replication to chronic disease manifestations (Table 1) [23,35]. The virus exhibits tropism for fibroblasts in the skin, muscle and joints in both humans and permissive mouse models [36]. In vitro assays using human (MRC5) and murine (Hs789.SK) fibroblasts have demonstrated a significantly higher susceptibility to infection when compared to other cell types, such as macrophages, suggesting that fibroblasts may enter a state of “hyperpermissivity” to CHIKV [25].

Beyond supporting viral replication, fibroblasts actively sense infection and initiate innate responses; however, CHIKV modulates these pathways to facilitate its persistence. In human synovial fibroblasts, CHIKV suppresses NF-κB by downregulating the adaptor proteins TRAF6, IRAK1 and IRAK2, weakening the antiviral response and promoting viral replication. Molecularly, this effect is mediated by the induction of miR-146a, which negatively regulates these proteins, maintaining NF-κB in a suppressed state, decreasing the production of pro-inflammatory cytokines and creating an environment conducive to viral persistence and chronic disease manifestations [37]. Nevertheless, the molecular mechanisms governing CHIKV tropism for fibroblasts and their marked susceptibility to infection remain to be fully elucidated.

Similar to muscle cells, dermal fibroblasts can survive infection while maintaining viral RNA for more than 100 days, thereby acting as long-term viral reservoirs. This persistence is accompanied by sustained production of inflammatory mediators, which is thought to underlie chronic arthralgia following acute disease [23]. During CHIKV infection, synovial fibroblasts act as key amplifiers of inflammation [38] by secreting high levels of IL-6 and establishing a pro-inflammatory microenvironment that recruits CD14^+^ monocytes and promotes their differentiation into osteoclast-like macrophages. This process is further driven by the IL-6-RANKL-OPG axis, a pivotal regulator of inflammatory bone remodeling [39,40,41]. Within this pathway, IL-6 upregulates RANKL expression while downregulating its decoy receptor, osteoprotegerin (OPG), thereby enhancing osteoclastogenesis and bone resorption [39,41]. The resulting imbalance in the RANKL/OPG ratio, together with elevated CCL2/MCP-1 and prostaglandin E2 signaling [38], contributes to the accumulation of osteoclast precursors and the release of arthritogenic mediators that drive joint pain and structural damage in the CHIKV-infected synovium. Collectively, these findings underscore the pro-inflammatory crosstalk between fibroblasts, macrophages and osteoclasts in the infected joint, which mirrors mechanisms described in rheumatoid arthritis, reinforcing the pathogenic relevance of the IL-6-RANKL-OPG axis in CHIKV-induced chronic arthropathy [41].

## 4. Macrophages and Monocytes in CHIKV Infection: Roles in Inflammation, Viral Persistence and Dissemination

Macrophages are also susceptible to CHIKV [25,42] and are primarily associated with the tissue inflammation at infection sites (Table 1) [43]. Histopathological analyses from CHIKV-infected individuals have demonstrated that these cells are abundant in the skin during the acute phase of the disease [43]. Moreover, in a murine model of virus-induced arthritis and myositis, treatment with bindarit, an inhibitor of CCL2/MCP-1 production, impaired the macrophage migration and significantly reduced muscle and joint inflammation [32]. This effect is likely related to the ability of macrophages to respond to CHIKV infection by producing GM-CSF, TNF-α and IL-6 [42].

Although the contribution of macrophages to inflammation during CHIKV infection is well-documented, their role is not limited to this function. In a mouse model, depletion of macrophages by clodronate resulted in prolonged viremia, indicating the participation of these cells in viral clearance [44]. Additionally, experiments using CCR2^−/−^ mice demonstrated that macrophages recruited to the infected site limit neutrophil accumulation, thereby preventing excessive tissue damage [45]. Furthermore, macrophages with an anti-inflammatory M2 phenotype were also identified in the musculoskeletal tissue of CHIKV-infected mice [46], and the loss of these cells, evidenced by downregulation of markers such as TGF-β, Ym1 and Msr1, was associated with exacerbated inflammation, suggesting that M2 macrophages play a central role in modulating the inflammatory response. These findings indicate that macrophages serve as targets for infection, promote viral elimination and are important both for modulating the inflammatory response and for the development of rheumatic disease.

A growing body of evidence also suggests that macrophages, as seen for fibroblasts, can also act as significant viral reservoirs during the late phases of CHIKV infection, directly contributing to the persistence of clinical manifestations [42,47,48]. In a patient from Réunion Island with chronic infection, CHIKV RNA was detected in synovial macrophages 18 months after infection [28]. This finding is corroborated by studies in non-human primates, where the virus was identified in macrophages in the liver, spleen, joints and lymphoid organs up to 3 months after experimental infection [48]. CHIKV persistence in these myeloid cells appears to involve phagocytosis of infected apoptotic bodies, a mechanism that allows infection of macrophages in a dormant, non-inflammatory form [47], together with the virus’s ability to modulate intracellular processes in these cells by inhibiting apoptosis and suppressing antiviral gene expression, including IFN-α4 and ISG56 [42].

The permissiveness of circulating monocytes is more controversial: although early in vitro work suggested that primary monocytes are not permissive [25], more recent research has shown that monocytes can become infected, respond rapidly by secreting chemokines and cytokines (e.g., IL-8/CXCL8, IL-12, CCL5/RANTES, CXCL10/IP-10 and CCL4/MIP-1β) [39]. Ly6C^+^ monocytes recruited to the skin during early CHIKV infection support CHIKV infection and are implicated in viral dissemination to draining lymph nodes (dLNs), which likely enables the viral spreading into the blood and to distal tissue sites [49] (Figure 1). In a recent study on fatal outcomes of Chikungunya disease (CHIKD), it was demonstrated that CHIKV invades the CNS by infecting CD14^+^CD16^+^ monocytes which, in the presence of high levels of CCL2, migrate through the blood–brain barrier into the brain parenchyma, a mechanism known as Trojan Horse strategy [50].

Acute CHIKV infection in pediatric patients was shown to elicit a strong monocyte-driven innate response, particularly characterized by the expansion of intermediate CD14^++^CD16^+^ monocytes and an activated subset of CD14^+^ monocytes. These monocytes, as well as DCs, exhibited elevated expression of the CHIKV envelope protein E2 during the acute phase, suggesting that these cell populations are targets and/or carriers of viral antigen [51]. Experimental infection studies using alphaviruses such as Semliki Forest virus and West Nile virus have shown that, following cutaneous infection, Langerhans cells become activated, undergo phenotypic changes and migrate to draining lymph nodes carrying the virus [52,53]. Although direct experimental evidence for CHIKV is still lacking, these findings from related viruses indicate that dissemination of CHIKV via Langerhans cells represents a plausible route for viral spread to secondary lymphoid organs [54]. Recent findings also demonstrated early accumulation of CHIKV RNA in MARCO-expressing lymphatic endothelial cells (LECs) within dLNs in the first 24 h post-infection, pointing to an alternative route of viral entry into lymph nodes [55] (Figure 1).

## 5. Dendritic Cell Dynamics During CHIKV Infection

Peripheral blood circulating DCs are detected in CHIKV-infected patients [24]. Also, frequencies of plasmacytoid DCs (pDCs), myeloid DCs (mDCs), non-plasmacytoid/non-myeloid DCs and monocytes/macrophages were shown to be increased in the peripheral blood of adult Rhesus macaques after experimental CHIKV infection, but aged animals displayed a delayed expansion of mDCs [56]. In cynomolgus macaques, early infection was marked by increased proliferation of monocytes, later followed by pDCs, whereas mDCs initially exhibited reduced proliferation that quickly normalized. These dynamics were reflected in circulating cell counts [57]. pDCs, specialized producers of IFN-I [58], are not permissive to CHIKV infection but act as key sensors that detect viral components or infected cells and mount an IRF7-dependent production of IFN-I (Table 1). This early pDC IRF7-mediated response not only suppresses viral spread in vivo but also amplifies natural killer (NK) cell activity [59].

Although robust studies on the permissiveness of conventional dendritic cells to CHIKV are still scarce, it has been established that these cells can internalize viral antigens without supporting productive infection, thereby acting as a bridge between innate and adaptive immunity through antigen presentation to T lymphocytes [60] (Table 1). A study using purified CHIKV capsid protein demonstrated DC maturation with upregulation of the costimulatory molecule CD86 and secretion of TNF-α and IL-12p70, ultimately enhancing CD4^+^ T cell activation [61]. The role of cDC1 cross-presentation has been further established in Batf3- or Wdfy4-deficient mice (critical regulators of cDC1 development), in which epitope-specific CD8^+^ T cell responses were impaired despite unaffected viral burdens in joint tissues and spleen [62].

CHIKV infection also induces the accumulation of CD11c^+^CD11b^+^ DCs at the initial infection site, with a concomitant reduction in DC immunoreceptor (DCIR) surface expression, suggesting receptor internalization and signaling alterations [62]. DCIR deficiency results in dysregulated cytokine responses, with enhanced IL-6 and IL-10 and reduced IL-12 production by DCs and in vivo DCIR^−/−^ mice that developed earlier and more severe pathology, including pronounced edema and tissue damage, despite similar early viral replication. At later stages, elevated viral loads in DCIR^−/−^ mice correlated with exacerbated pathology. These findings identify DCIR as a negative regulator of CHIKV immunopathogenesis, acting to limit excessive inflammation while maintaining sufficient antiviral defense [63]. Importantly, this regulatory role was not observed for other C-type lectin receptors, such as mouse DC-SIGN and SIGNR3 [63].

## 6. The Ambiguous Role of Neutrophils in CHIKV Immunity and Pathogenesis

The role of neutrophils in arboviral infections has been under increasing investigation [64]. To date, however, there is a lack of consolidated evidence regarding the susceptibility of these cells to CHIKV infection. Nevertheless, a growing body of research suggests that neutrophils may play a pivotal role during the acute phase of the disease and in determining the severity of arthritic symptoms. Experiments conducted in mice deficient in CCR2, IL-17A, or TLR3 have shown that dysregulated neutrophil recruitment results in exacerbated inflammation and cartilage lesions, reinforcing the contribution of these leukocytes to the pathogenesis of CHIKV-induced arthritis [45,65,66]. In IFN-β-deficient mice, neutrophil depletion leads to a significant reduction in joint inflammation without substantially altering viral replication, indicating that neutrophils are key determinants of infection-associated tissue damage but do not play a central role in controlling viremia. Furthermore, neutrophil infiltration into draining lymph nodes has been shown to compromise lymphoid architecture and impair the development of the adaptive humoral immune response, negatively affecting CHIKV-specific viral clearance mechanisms [67].

Conversely, a study using an experimental infection model in zebrafish identified neutrophils as a key cell population involved in the production of IFN-I and the regulation of CHIKV replication [68]. Additionally, neutrophils have been shown to respond to CHIKV infection by releasing extracellular traps (NETs) through ROS- and TLR7-dependent mechanisms. Research showed that NETs were able to capture and neutralize CHIKV particles in vitro, and this antiviral activity was abolished by DNase treatment (Table 1). Further, in vivo assay demonstrated that IFNAR^−/−^ mice treated with DNase showed increased viral load and earlier mortality compared to untreated controls, providing evidence for the role of NETs in limiting CHIKV replication systemically. In acutely infected patients, circulating NET levels positively correlate with viral load, supporting its relevance in natural human infection [33]. Overall, these findings establish NETosis as an innate antiviral mechanism in CHIKV acute infection, contributing to early viral control.

Thus, neutrophils can be said to play an ambiguous role during CHIKV infection. While early NETosis exerts a protective effect, excessive or dysregulated neutrophil recruitment can amplify inflammation, compromise lymphoid architecture and promote tissue damage. This dual role underscores the importance of understanding the signals that balance protective versus harmful neutrophil responses and their impact on disease chronicity, which remains to be fully elucidated.

## 7. Cytotoxic Innate Cells in CHIKV Control

Acute CHIKV infection is associated with a high frequency of NK cells in peripheral blood [28,34], which play a pivotal role in controlling the infection [69]. Phenotypic and functional analyses of NK cells from CHIKV-infected patients indicate that, during the acute phase, these cells are strongly activated (Table 1), with increased expression of CD69 and HLA-DR markers [34]. This activation is persistent and prolonged, in contrast to other arboviral infections, such as Dengue virus, which induce only transient NK cell activation [69]. As the infection progresses, the NK cell repertoire shifts toward a mature phenotype (CD56^dim^/CD57^+^), exhibiting high cytotoxic potential but reduced capacity for IFN-γ production [34,70]. Clinical studies show that individuals in the acute or convalescent phase have elevated numbers of NK cells, with an increased proportion of cytotoxic NK cells (perforin^+^CD3^−^CD56^+^) in both phases. In contrast, individuals with chronic infection exhibit a reduced population of perforin^+^ NK cells, accompanied by increased expression of inhibitory receptors NKG2A and CD94 [71]. These findings underscore the critical role of NK cells in directly eliminating viral reservoirs.

NK cells constitute the second most abundant cell type in the paw pads of acute CHIKV infection in mice [44]. Moreover, the extent of inflammatory damage is directly proportional to the activity of these cells [72]. Clinical data have also shown the presence of NK cells in the synovial tissue of patients who developed chronic arthralgia. These cells were observed in close proximity to macrophages persistently infected with the virus and exhibited an activated phenotype (NKRp44^+^) [28]. Significantly elevated serum levels of granzyme A were detected in both CHIKV-infected patients and experimental animals, correlating with viral load and disease severity [73]. These findings suggest that, while NK cells are essential for controlling infection, they also contribute to a persistent antiviral response, which may be associated with the maintenance of joint inflammation and the development of chronic arthralgia.

A subset of αβ T cells known as NKT-like cells expresses NK activation receptors and has a highly specialized effector memory function. NKT-like cells from patients in the acute and convalescent stages of CHIKV infection were phenotypically and functionally analyzed. It was found that NKT-like cells from chikungunya patients expressed more activating receptors than controls, and the frequency of IFN-γ^+^ NKT-like cells was higher in the convalescent stage, indicating an early and effective antiviral response [74]. The profile and function of NKT-like cells were evaluated in a cross-sectional cohort consisting of chronic patients, recovered individuals and health controls. The authors demonstrated that the percentage of NKT-like cells was low in chronic patients, and these cells exhibited increased expression of the inhibitory receptors NKG2A and CD94. Moreover, IFN-γ and TNF-α expression on NKT-like cells was high in the chronic patients, suggesting a pathogenic role of NKT-like cells in chronic settings [71].

## 8. Immunomodulatory Effects of *Ae. aegypti* Saliva During CHIKV Infection

During blood-feeding, *Ae. aegypti* inoculates viral particles together with its saliva, which constitutes a complex mixture of vasoactive, anticoagulant and immunomodulatory molecules. These salivary components facilitate the mosquito’s feeding while simultaneously modulating viral transmission and influencing host susceptibility [75,76,77]. In the context of CHIKV infection, saliva-derived factors enhance viral replication, increase viremia and promote viral dissemination to peripheral organs, which is directly associated with disease severity [78].

Several *Ae. aegypti* salivary proteins have been shown to interact with human immune receptors [79]. Moreover, CHIKV infection positively modulates the expression of salivary proteins associated with viral survival, replication and transmission [80]. Among these, sialokinin, a vasodilatory peptide analogous to substance P, is one of the best characterized [81]. Sialokinin acts on the NK1R receptor (neurokinin-1), inducing nitric oxide production, thereby increasing vascular permeability and promoting leukocyte migration to the bite site. This recruitment enriches the local environment with neutrophils, whose accumulation triggers inflammation that retains the virus in the skin and simultaneously promotes the recruitment of virus-permissive cells, such as monocytes, thereby enhancing viral replication and increasing the infection efficiency [82,83,84,85].

Ex vivo and in vitro studies with isolated sialokinin or salivary gland extracts (SGEs) show that saliva components significantly reduce the expression of IFN-β and inducible nitric oxide synthase (iNOS) while increasing IL-10 expression and inhibiting the inflammasome in macrophages [85,86]. Furthermore, they suppress monocyte activation, as evidenced by the downregulation of genes related to IFN-I signaling, including IFIT2, IFI44L, SIGLEC1 and ACP5, as well as by the reduced expression of activation markers HLA-DR, CD16 and CD169, effects that were also observed for MDMs [76]. Similarly, human fibroblasts infected with CHIKV and exposed to *Ae. aegypti* saliva show reduced expression of IFI6, IFI16, IFI27, IFI30 and phosphorylated STAT2, alongside significantly higher viral loads compared with fibroblasts infected in the absence of saliva [87]. These findings collectively suggest that, although salivary factors induce the recruitment of innate immune cells to the bite site, they concurrently alter the function of both resident and infiltrating cells, skewing the immune milieu toward a permissive phenotype. Supporting this, comparisons between CHIKV infection via needle-mediated and mosquito-borne CHIKV infection reveal that the latter suppresses IFN-γ and TLR3 expression at the bite site while inducing IL-4 expression [88]. Consistently, experiments comparing subcutaneous inoculation of CHIKV alone versus inoculation following bites from uninfected *Ae. aegypti* mosquitoes show that mosquito bites reshape the host’s early immune response by downregulating pro-inflammatory and antiviral genes such as TLR3, IFN-γ, TNF-α and IFN-β, while inducing IL-4 and IL-10. This establishes a microenvironment favorable to viral replication and correlates with enhanced viral dissemination and disease progression [78].

Consistent with these immunomodulatory effects, studies using *Ae. aegypti* SGE show that saliva does not alter the differentiation, maturation, or viability of bone marrow–derived DCs in vitro, but it significantly modulates the function of these cells in ex vivo splenocyte cultures by reducing the secretion of IL-12, a cytokine central to the activation of innate immune responses. These findings indicate that, although DCs survive the exposure to saliva, their functional capacity is affected, contributing to local immunomodulation at the bite site [89,90]. In addition, *Ae. aegypti* saliva exerts a dual effect on NK cells, reducing their total numbers in circulation while promoting transient increases in specific subsets, including NKT (CD3^+^CD56^+^) and NK (CD3^−^CD56^+^) cells, at distinct time points after the bite. These effects can persist for up to 7 days post-bite, altering the cytotoxic and inflammatory activity of these cells and potentially modulating the innate immune response during arboviral infections [91].

Together, these findings indicate that *Ae. aegypti* saliva plays a central role in shaping the early innate immune landscape during CHIKV infection, attenuating inflammatory and antiviral pathways, inducing leukocyte migration to the bite site (including neutrophils and monocytes) [82,83,84,85], modulating NK cell populations [91], impairing the activation and function of myeloid cells (monocytes, MDMs and DCs) [76,85,86,89,90] and non-myeloid cells (fibroblasts) [87] and fostering a microenvironment conductive to viral replication and dissemination [78,88]. Yet, despite growing evidence of these effects, specific salivary molecules and their molecular targets remain poorly characterized. Unraveling how these proteins interact with the innate immune components can be a key to understanding the early events of arboviral infection and may uncover novel targets for vector-based intervention strategies.

## 9. Conclusions and Future Directions

Chikungunya virus (CHIKV) remains a major global health challenge due to its capacity to elicit both protective and pathogenic innate immune responses that ultimately shape the course of infection. As highlighted in this review, different innate cell types, including fibroblasts and macrophages, not only support viral replication but also sustain inflammation, thereby contributing to both acute and chronic disease manifestations.

Despite considerable advances in understanding the roles of innate cells during CHIKV infection, critical questions remain unresolved. Elucidating the mechanisms that underlie viral persistence in fibroblasts and macrophages and their contribution to long-term tissue inflammation may prove central to mitigating chronic outcomes. Future studies integrating single-cell RNA sequencing and spatial transcriptomics of synovial and muscle tissues from both acute and chronic CHIKV patients could uncover pathogenic cellular subsets and their molecular signatures. Similarly, multi-omics profiling of patient-derived immune cells could be a promising tool for clarifying the metabolic and transcriptional programs that distinguish antiviral from pro-inflammatory phenotypes.

The dual, often contradictory, roles of neutrophils in protection versus pathology require further clarification. Controlled in vitro culture systems or murine models using neutrophil-specific gene knockouts could help dissect the pathways that shift neutrophil function from protective to pathological. Therefore, shedding light on the role of innate immunity during the transition from acute to chronic disease. In parallel, there is a growing need to evaluate the therapeutic viability of modulating inflammatory pathways driven by cytokines such as IL-6 and TNF-α. Targeted approaches that fine-tune their activity in specific cell populations or disease phases may prove more beneficial than broad neutralization. Controlled, short-term inhibition during the post-acute phase could help attenuate joint inflammation and tissue damage while preserving antiviral response.

Future research employing longitudinal patient cohorts will be essential to delineate the cellular and molecular pathways that govern the balance between viral clearance and chronic inflammation and to elucidate the role of innate cells during chronic symptoms. Such insights may ultimately guide the development of host-directed therapies and targeted immunomodulatory interventions to reduce disease burden and improve clinical outcomes.

Additionally, *Ae. aegypti* saliva acts as an early modulator of innate immunity during CHIKV infection. Many salivary proteins remain poorly characterized, and their effects on various innate immune cells are still not well understood. Studying these interactions in experimental models mimicking natural infection is essential to integrate vector-mediated immunomodulation into the design of effective antiviral and vaccine strategies.

Altogether, a deeper understanding of how innate immunity balances antiviral defense and tissue integrity would be crucial not only for unraveling disease mechanisms but also for advancing the rational design of effective immunomodulatory and antiviral therapeutic strategies.

## Figures and Tables

**Table 1:** Summary of the susceptibility and functional roles of major innate immune cell types during CHIKV infection.

Cell Type	Productively Infected	Activated	Main Immune Function(s)
Fibroblasts	Yes	Yes	Major viral replication site; produce cytokines and chemokines; act as long-term reservoirs contributing to chronic inflammation.
Macrophages	Yes	Yes	Participate in viral clearance; secrete inflammatory mediators; may harbor persistent viral RNA during chronic phase.
Monocytes	Unclear	Yes	Produce cytokines and chemokines; contribute to viral dissemination and CNS entry via the “Trojan horse” mechanism.
Langerhans cells	Unclear	Yes (antigen carriers)	Likely involved in viral transport to draining lymph nodes.
Plasmacytoid DCs (pDCs)	No	Yes	Detect infected cells and produce large amounts of type I interferons.
Conventional DCs (cDCs)	Unclear	Yes	Present antigens to T cells and bridge innate and adaptive responses.
Neutrophils	No	Yes	Release extracellular traps (NETs) with antiviral effects; excessive recruitment contributes to tissue damage.
NK and NKT-like cells	No	Yes	Contribute to infection control; persistent and prolonged activation; high cytotoxic potential; cause tissue damage; pathogenic role during the chronic phase.

## Data Availability

No new data were created.

## Abbreviations

The following abbreviations are used in this manuscript:

CHIKD	Chikungunya Disease
CHIKV	Chikungunya Virus
CXCL8	C-X-C Motif Chemokine Ligand 8
DCIR	Dendritic Cell Immunoreceptor
DCIR^−^/^−^	DCIR Knockout Mice
DC-SIGN	Dendritic Cell-Specific Intercellular Adhesion Molecule-3-Grabbing Non-Integrin
DCs	Dendritic Cells
DNase	Deoxyribonuclease
E2	CHIKV Envelope Protein E2
GM-CSF	Granulocyte-Macrophage Colony-Stimulating Factor
HLA-DR	Human Leukocyte Antigen–DR Isotype
IFN-I	Type I Interferon
IFN-β	Interferon Beta
IFN-γ	Interferon Gamma
IFNAR^−^/^−^	Type I Interferon Receptor Knockout Mice
IL-6	Interleukin 6
IL-8	Interleukin 8
IL-10	Interleukin 10
IL-12	Interleukin 12
IL-12p70	Interleukin 12p70
IL-17A	Interleukin 17A
iNOS	Inducible Nitric Oxide Synthase
IRF7	Interferon Regulatory Factor 7
ISG56	Interferon-Stimulated Gene 56
Ly6C^+^	Lymphocyte Antigen 6 Complex, Locus C Positive (mouse monocyte marker)
M2	Alternatively Activated (anti-inflammatory) Macrophage Phenotype
MARCO	Macrophage Receptor with Collagenous Structure
MRC5	Human Fetal Lung Fibroblast Cell Line
Msr1	Macrophage Scavenger Receptor 1
mDCs	Myeloid Dendritic Cells
NKG2A	NK Cell Inhibitory Receptor
NKRp44^+^	NK Cell Activating Receptor Marker
NK cells	Natural Killer Cells
NKT-like	Natural Killer T-like Cells
NETs	Neutrophil Extracellular Traps
NF-κB	Nuclear Factor Kappa B
OPG	Osteoprotegerin
pDCs	Plasmacytoid Dendritic Cells
RANKL	Receptor Activator of Nuclear Factor Kappa-B Ligand
RNA	Ribonucleic Acid
ROS	Reactive Oxygen Species
SGE	Salivary Gland Extract
SIGNR3	SIGN-related 3 (mouse homolog of DC-SIGN)
TGF-β	Transforming Growth Factor Beta
TNF-α	Tumor Necrosis Factor Alpha
TRAF6	TNF Receptor-Associated Factor 6
TLR3	Toll-Like Receptor 3
TLR7	Toll-Like Receptor 7
Wdfy4	WD Repeat and FYVE Domain Containing 4 (critical for cDC1 development)
Ym1	Chitinase-Like Protein Ym1 (mouse marker for M2 macrophages)
